# Effects of Lysine Cell Mass Supplementation as a Substitute for L-Lysine·HCl on Growth Performance, Diarrhea Incidence, and Blood Profiles in Weaning Pigs

**DOI:** 10.3390/ani11072092

**Published:** 2021-07-14

**Authors:** Jinsu Hong, Hee-Seong Kim, Sungho Do, Hong-Jun Kim, Sung-Won Kim, Sung-Kwon Jang, Yoo-Yong Kim

**Affiliations:** 1 Department of Agricultural Biotechnology and Research Institute of Agriculture and Life Science, Seoul National University, Seoul 08826, Korea; jinsu.hong@sdstate.edu (J.H.); Heeseong@ksu.edu (H.-S.K.); sunghod2@illinois.edu (S.D.); chriskim11@naver.com (H.-J.K.); kisw808@naver.com (S.-W.K.); 2 Department of Animal Science, South Dakota State University, Brookings, SD 57007, USA; 3 Daesang Corporation, Seoul 02154, Korea; sungkwon.jang@daesang.com

**Keywords:** lysine cell mass, growth performance, blood profiles, diarrhea incidence, weaning pigs

## Abstract

**Simple Summary:**

Lysine cell mass (LCM) is a potential protein and lysine source for pigs. However, the potential value of LCM as a lysine source for the swine diet has not been investigated. Therefore, this study was conducted to evaluate the effects of LCM as an alternative lysine source in diets for weaning pigs. In the first experiment, an increase in dietary LCM from 0 to 1% did not affect the growth performance and diarrhea incidence of weaning pigs. However, in the second experiment, increasing the level of LCM supplementation for replacing L-lysine·HCl from 0% to 100% quadratically decreased the growth performance of weaning pigs such that replacing 0 to 80% of L-lysine·HCl with LCM had no difference in the growth performance, whereas LCM supplementation with 100% replacement of L-lysine·HCl decreased the average daily gain and gain to feed ratio of weaning pigs. We concluded that LCM could be included in the diet for weaning pigs as a substitute of L-lysine·HCl up to 2.8% and 1.76% for phase 1 and phase 2, respectively, without negative impacts on the performance of weaning pigs.

**Abstract:**

This study was conducted to evaluate the effects of lysine cell mass (LCM) as an alternative lysine source in diets for weaning pigs on growth performance, diarrhea incidence, and blood profiles. In experiment 1, a total of 200 weaning pigs, with an average body weight (BW) of 6.89 ± 1.04 kg, were allotted into one of five treatments with four replicates of 10 pigs per pen in a randomized complete block design (RCBD). The dietary treatments were composed of LCM supplementation (0, 0.25, 0.5, 0.75, or 1.0%) with partial replacement of L-lysine·HCl (0 to 0.8% for phase 1 diets and 0 to 0.07% for phase 2 diets). The BW and feed intake were recorded at the end of each phase (d 0 to 14 for phase 1, d 14 to 35 for phase 2), and diarrhea incidence was checked daily throughout the experimental period. Blood samples were taken from the jugular vein of pigs at 2 weeks and 5 weeks to determine the blood profiles of weaning pigs. In experiment 2, a total of 144 weaning pigs with an average BW of 6.44 ± 1.19 kg were allotted into one of six treatments with six replicates of four pigs per pen in RCBD. The dietary treatments were composed of LCM supplementation (0 to 3.5% for phase 1 diets and 0 to 2.2% for phase 2 diets) with replacement of L-lysine·HCl from 0 to 100%. In experiment 1, partial replacement of L-lysine·HCl with 0 to 1% LCM did not affect growth performance and diarrhea incidence of pigs. An increase in the LCM supplementation from 0 to 1% with partial replacement of L-lysine·HCl had no influence on the blood urea nitrogen concentrations, whereas it resulted in a linear decrease (*p* < 0.05) in the serum IgG concentrations for 5 weeks. In experiment 2, increasing the dietary level of LCM with replacement of L-lysine·HCl quadratically decreased (*p* < 0.05) ADG and G–F ratio for phase 2 and G–F ratio for the overall period such that 100% replacement of L-lysine·HCl with LCM decreased ADG and G–F ratio of weaning pigs. An increase in the LCM supplementation with replacement of L-lysine·HCl tended to decrease linearly (*p* < 0.10) the diarrhea incidence of weaning pigs for the overall period and linearly decrease (*p* < 0.05) the serum IgG concentrations for 2 weeks. In conclusion, partial replacement of L-lysine·HCl with LCM from 0 to 1% had no negative impacts on the growth performance, but 100% replacement of L-lysine·HCl with LCM decreased the growth performance of weaning pigs. Therefore, LCM could be included in the diets for weaning pigs up to 2.8% and 1.76% for phase 1 and phase 2, respectively, as a substitute for L-lysine·HCl without detrimental effects on the performance of weaning pigs.

## 1. Introduction

Feed cost is considered the most crucial part of swine production because it accounts for approximately 50 to 60% of total production costs [1,2]. Since highly digestible ingredients are used in feed as protein sources for weaning pigs, the feed cost in the weaning diet is more expensive than growing–finishing diets [3,4]. Moreover, a low-protein diet supplemented with synthetic amino acids has been applied to the diet for weaning pigs to reduce the feed cost, diarrhea, and total nitrogen content of pig manure, resulting in an increased use of the synthetic amino acids [5,6,7,8]. Severe climate changes, such as drought, flood, and trade issues between countries have influenced the market prices of protein ingredients and synthetic amino acids. To prevent fluctuations in the market price, there is a need to find alternative feedstuff for protein ingredients and synthetic amino acids to be used in the diets of weaning pigs.

The most commonly used synthetic lysine is L-lysine·HCl, produced by the industry in large amounts using bacterial fermentation. After fermentation with microorganism and carbohydrate sources, the products go through cell separation to divide into lysine products and cell sludge. The dried bacterial cell mass produced from the cell sludge is called the lysine cell mass (LCM). LCM has a high protein content (65–75%; [9]) and is rich in amino acids, such as glutamine 8.05–8.55% and lysine 2.36–8.23% [10,11].

LCM has great potential to be used as an alternative high protein source and lysine source for monogastric animals. Some studies have evaluated the effect of LCM as a protein source in weaning pigs [10], growing pigs [9,11], and broiler chickens [12]. However, the potential value of LCM as a lysine source for the swine diet has not been investigated. Therefore, this experiment was designed to evaluate the effect of LCM as a substitute for L-lysine·HCl in weaning pigs’ diets on growth performance, diarrhea incidence, and blood profiles.

## 2. Materials and Methods

All experimental procedures involving animals were approved and conducted by the Animal Experimental Guidelines provided by the Institutional Animal Care and Use Committee at Seoul National University (SNU-161004-1).

### 2.1. Lysine Source

The L-lysine·HCl (78%) and LCM used in the current study were provided by Daesang corporation (Seoul, Korea). Briefly, after pure L-lysine was removed from the *Corynebacterium glutamicum* fermentation medium, the live bacteria were killed at high temperature, and then the remaining medium was dried to produce LCM. Glucose, raw sugar, and molasses were used as carbon sources for *Corynebacterium glutamicum* fermentation. The analyzed chemical composition of LCM is presented in Table 1.

### 2.2. Experiment 1

#### 2.2.1. Animals and Housing

A total of 200 crossbred pigs (initial body weight (BW) of 6.89 ± 1.04 kg; Large White Yorkshire-Landrace female × Duroc male) weaned at the age of 28 days were used for experiment 1. All pigs were housed in slotted plastic floor pens each equipped with a feeder and a nipple waterer throughout the weaning period (0 to 5 weeks). All pigs were allowed access to feed and water *ad libitum* throughout the experimental period. The temperature in the nursery room was maintained at 30 °C in the first week, and it decreased by 1 °C every week, so that it was 26 °C in the 5th week.

#### 2.2.2. Experimental Diet

Five experimental diets included a corn–soybean meal (SBM)-based basal diet with L-lysine·HCl replaced by 0.25, 0.50, 0.75, or 1.0% of LCM. The experimental diets were fed in 2 phases: phase 1 for 14 d and phase 2 for 21 d. A proportion of L-lysine·HCl from 0 to 0.08% was replaced by 0 to 1.0% LCM for the phase 1 diet, and 0 to 0.07% of the L-lysine·HCl was replaced by 0 to 1.0% LCM for the phase 2 diet. The experimental diets were formulated to have similar levels of metabolizable energy (ME), crude protein (CP), calcium, total phosphorus, and total Lys, Met, and Thr contents. The experimental diets were formulated to meet the CP requirements of the National Research Council (NRC; [13]) and to meet and exceed the NRC [14] nutrient recommendations for weaning pigs. The formulas and chemical compositions of the experimental diets are presented in Table 2 and Table 3.

#### 2.2.3. Experimental Design and Procedure

The five experimental diets were allotted to the 20 pens with 10 pigs per pen (5 barrows and 5 gilts in each pen) in a randomized complete block design by the experimental animal allotment program (Kim and Lindemann [15]) with a balance of initial BW and sex. A previous study within the facility used for experiment 1 observed average coefficients of variation of less than 5.0 for measured parameters of ADG and ADFI with the same replications and the number of pigs per pen.

The BW and feed intake were recorded at the end of each phase to calculate the average daily gain (ADG), average daily feed intake (ADFI), and gain-to-feed ratio (G–F ratio). The fecal score was checked daily at 0900 throughout the weaning period (0–5 weeks). Fecal score was assessed on a pen basis by using the following fecal scoring system: 1 = firm and shaped feces, 2 = normal and formed feces, 3 = soft and wet feces, 4 = mild diarrhea, and 5 = watery diarrhea. After recording the data, the evidence of diarrhea on the pigs and feces on the floor were cleaned away to separate new observations from previous observations.

Blood samples were taken from the jugular vein of 6 selected pigs per treatment with average BW of each treatment after 3 h of fasting at the end of each phase (2 weeks and 5 weeks). Blood samples were collected in serum tubes (SST^TM^Ⅱ Advance, BD Vacutainer, Becton Dickinson, Plymouth, UK) and ethylenediaminetetraacetic acid (EDTA) tubes (K_2_E, BD Vacutainer, Becton Dickinson, Plymouth, UK), respectively. The collected samples were centrifuged at 1957× *g* and 4 °C for 15 min (Eppendorf centrifuge 5810R, Eppendorf, Hamburg, Germany) after clotting at room temperature for 30 min. The supernatants were carefully transferred to microtubes (Axygen, Union City, CA, USA) and stored at −20 °C in a freezer for later determination of serum concentrations for cortisol, insulin-like growth factor-1 (IGF-1), insulin, immunoglobulin A (IgA), and immunoglobulin G (IgG), and of plasma concentration for blood urea nitrogen (BUN).

### 2.3. Experiment 2

#### 2.3.1. Animals and Housing

A total of 144 crossbred pigs (initial BW of 6.44 ± 1.19 kg; Large White Yorkshire-Landrace female × Duroc male) weaned at the age of 28 days were used for experiment 2. All pigs were housed in slotted plastic floor pens each equipped with a feeder and a nipple waterer throughout the weaning period (0 to 5 weeks). All pigs were allowed access to feed and water *ad libitum* throughout the experimental period. The temperature in the nursery room was maintained at 30 °C in the first week, and it decreased by 1 °C every week, so that it was 26 °C in the 5th week.

#### 2.3.2. Experimental Diet

Six experimental diets included corn–SBM-based basal diets with L-lysine·HCl replaced by LCM. The experimental diets were fed in 2 phases: phase 1 for 14 d and phase 2 for 21 d. A proportion of L-lysine·HCl from 0.27 to 0% was replaced by 0 to 3.5% of LCM for the phase 1 diet, and 0.18 to 0% of the L-lysine·HCl was replaced by 0 to 2.2% of LCM for the phase 2 diet. The experimental diets were formulated to have similar levels of ME, CP, calcium, total phosphorus, and total Lys, Met, and Thr contents. The experimental diets were formulated to meet the CP requirements of the NRC [13] and to meet and exceed the NRC [14] nutrient recommendations for weaning pigs. The formulas and chemical compositions of the experimental diets are presented in Table 4 and Table 5.

#### 2.3.3. Experimental Design and Procedure

The six experimental diets were allotted to the 36 pens with 4 pigs per pen (2 barrows and 2 gilts in each pen) in a randomized complete block design by the experimental animal allotment program (Kim and Lindemann [15]) with a balance of initial BW and sex. A previous study within the facility used for experiment 2 observed average coefficients of variation of less than 5.0 for measured parameters of ADG and ADFI with the same replications and the number of pigs per pen. The BW and feed intake were recorded at the end of each phase to calculate the ADG, ADFI, and G–F ratio. The incidence of diarrhea was checked daily at 09:00 throughout the whole experimental period. A diarrhea incidence score of 0 to 4 was given by counting pigs showing evidence of watery diarrhea. After recording the data, the evidence of diarrhea was cleaned away by wiping the evidence of the feces on the pigs to separate new observations from previous observations.

At the end of each phase, one pig (per pen) with a BW that was close to the average BW of pigs in that particular pen was selected. Blood samples were taken from the jugular vein of the selected pigs after 3 h of fasting. Blood samples were collected in serum tubes (SST^TM^Ⅱ Advance, BD Vacutainer, Becton Dickinson, Plymouth, UK) and EDTA tubes (K_2_E, BD Vacutainer, Becton Dickinson, Plymouth, UK), respectively. The collected samples were centrifuged at 1957× *g* and 4 °C for 15 min (Eppendorf centrifuge 5810R, Eppendorf, Hamburg, Germany) after clotting at room temperature for 30 min. The supernatants were carefully transferred to microtubes (Axygen, Union City, CA, USA) and stored at −20 °C in a freezer for later determination of serum concentrations for cortisol, IGF-1, insulin, IgA, and IgG, and of plasma concentration for BUN.

### 2.4. Sample Analysis

The experimental diets were ground into 1 mm particles by a Wiley mill (Wiley Mill Intermediate; Thomas Scientific, Swedesboro, NJ, USA). The LCM and experimental diets were analyzed for dry matter (procedure 967.03; [16]), crude ash (procedure 923.03; [16]), and nitrogen (N) by using the Kjeldahl procedure with Kjeltec (KjeltecTM 2200, Foss Tecator, Hilleroed, Denmark) and crude protein (N × 6.25; procedure 981.10; [16]). For determination of the AAs content in the LCM and diets, the samples were hydrolyzed at 110 °C for 24 h with 5 mL of 6 *N* hydrochloric acid per 20 mg sample. In the case of sulfur-containing amino acids, performic acid was used as a reagent for oxidation at the same level as hydrochloric acid. After acid hydrolysis, the hydrolysates were analyzed by the Beckman 6300 AA Analyzer (Beckman Instruments Corp., Palo Alto, CA, USA) using ninhydrin reagent and the hydrolysate program.

The concentration of BUN was analyzed by kinetic UV assay (Modular Analytics, Hitachi, Japan), and cortisol was analyzed using the γ-counter (Cobra 5010 Quantum model, Packard, USA) by the radioimmunoassay (RIA) method. The concentration of IGF-1 was analyzed by the chemiluminescent immunoassay (CLIA) method (Liaison XL model, Diasorin, Saluggia, Italy), and insulin was analyzed by the electrochemiluminescence immunoassay (ECLIA) method (Modular E model, Modular Analytics, Hitachi, Tokyo, Japan). The serum concentrations for IgG and IgA were determined by enzyme-linked immunosorbent assay (ELISA) according to the manufacturer’s guidelines (ELISA Starter Accessory Package, Pig IgG ELISA Quantitation Kit, Pig IgA ELISA Quantitation Kit; Bethyl, Montgomery, TX, USA). Samples were assayed in duplicates with a 1:20,000 (IgG) or 1:2000 (IgA) fold dilution. Plasma amino acids were analyzed by LC–MS/MS (3200 QTRAP, AB SCIEX, Framingham, MA, USA).

### 2.5. Statistical Analysis

All collected data were analyzed by least squares mean comparisons and evaluated with the general linear model (GLM) procedure of the SAS (SAS Institute Inc., Cary, NC, USA). The model included diet as the fixed effect and block as the random effect. Orthogonal polynomial contrasts were used to determine linear and quadratic effects by increasing the LCM supplementation levels. The pen was considered to be an experimental unit for growth performance, fecal score, and diarrhea incidence, and the individual pig was used as an experimental unit for blood profiles. For the diarrhea incidence (exp. 2), data of the number of pigs showing diarrhea were analyzed using the PROC FREQ procedure of the SAS. To test the hypotheses, *p* < 0.05 was considered significant. If pertinent, trends (0.05 ≤ *p* < 0.10) are also reported.

## 3. Results

The analyzed compositions of the LCM used in the present study is presented in Table 1. The Lys content in the LCM was 9.48%, and NPN was not detected. Lysine, Leu, and Arg were the most abundant indispensable AAs in the LCM, whereas His, Met, and Ile were the least abundant indispensable AAs in the LCM.

In experiment 1, the partial replacement of L-lysine·HCl with 0 to 1% LCM did not affect the ADG, ADFI, or G–F ratio of weaning pigs throughout the experimental period (Table 6). In addition, partial replacement of L-lysine·HCl with 0 to 1% LCM had no linear or quadratic influence on the fecal score of weaning pigs for the overall period (Table 7), whereas supplementation of LCM at 0.25% increased (*p* < 0.05) the fecal score compared to that of LCM 0.75% containing diet. An increase in LCM supplementation from 0 to 1% with partial replacement of L-lysine·HCl did not affect the blood concentrations of BUN and IGF-1 in weaning pigs (Table 8). However, increasing the supplementation level of LCM from 0 to 1% with partial replacement of L-lysine·HCl linearly increase (*p* < 0.05) the serum cortisol concentrations for 5 weeks and tended to linearly increase (*p* < 0.10) the serum insulin concentrations. An increasing level of dietary LCM from 0 to 1% with partial replacement of L-lysine·HCl resulted in a linear decrease (*p* < 0.05) in the serum IgG concentrations for 5 weeks, whereas it did not affect the serum IgA concentrations in weaning pigs.

**Figure 1:**
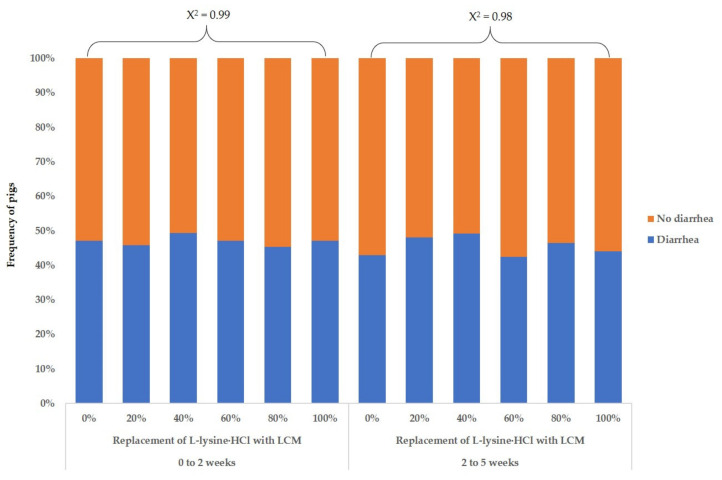
Effects of LCM supplementation levels on the frequency of diarrhea pigs (exp. 2).

In experiment 2, increasing the dietary level of LCM with replacement of L-lysine·HCl quadratically decreased (*p* < 0.05) ADG and G–F ratio for 2 to 5 weeks and G–F ratio for the overall period such that replacement of L-lysine·HCl from 0 to 80% with LCM did not affect the ADG and G–F ratio, whereas replacement of L-lysine·HCl at 100% with LCM decreased the ADG and G–F ratio of weaning pigs (Table 9). However, increasing the supplementation level of LCM with the replacement of L-lysine·HCl did not affect the ADFI of weaning pigs. An increase in the LCM supplementation with replacement of L-lysine·HCl did not affect (χ^2^ > 0.05) the frequency of the number of diarrhea pigs for 0 to 2 weeks or 2 to 5 weeks (Figure 1). In blood profiles (Table 10), replacement of L-lysine·HCl from 0 to 100% with LCM quadratically affected (*p* < 0.05) the concentration of BUN for 2 weeks such that the BUN levels increased when dietary LCM was increased from 0 to 60% replacement of L-lysine·HCl and then decreased when dietary LCM was further increased to 100% replacement of L-lysine·HCl. Increasing the dietary level of LCM with replacement of L-lysine·HCl linearly increased (*p* < 0.05) the concentrations of BUN for 5 weeks. Replacement of L-lysine·HCl with LCM did not affect the serum concentrations of IGF-1 or insulin, whereas the serum cortisol concentrations for 2 weeks tended to be linearly decreased (*p* < 0.10) by the increasing dietary levels of LCM. The increasing level of dietary LCM with replacement of L-lysine·HCl from 0 to 100% resulted in a linear decrease (*p* < 0.05) in the serum IgG concentrations for 2 weeks, whereas it did not affect the serum IgA concentrations in weaning pigs.

## 4. Discussion

The CP content of LCM (72.1%) used in the current study was similar to the values for LCM that were reported by Piao et al. (75.2–75.4%; [12]) and Wang et al. (70.4%; [17]) or the values for single-cell protein (SCP) from lysine fermentation that were reported by Zhang et al. (74.4%; [10]) and Son and Kim (74.0%; [9]). The Lys content of LCM (9.46%) was higher than the values reported by Piao et al. (5.12–6.88%; [12]), Zhang et al. (8.75%; [10]), and Wang et al. (2.50%; [11]). The LCM is the byproduct of lysine production by bacterial fermentation (*Corynebacterium)*. It has a high protein content compared to the values reported for yeast, algae, and fungi [18,19]. The similar CP content in LCM used in the current study compared to that used in the other studies could be explained by the fact that the LCMs were produced by bacterial fermentation. On the other hand, LCM production is dependent on the species of bacteria and the different carbon sources used as substrates, which may cause different nutritional values for the LCM [19,20]. Also, Piao et al. [12] reported that different degrees of centrifugation intensity for harvesting bacteria cells result in different CP values and AA compositions. Thus, the differences in Lys values for the LCM between other studies and the present study could have been due to the different processing conditions or procedures used, which may have resulted in different nutritional properties related to the CP and AAs.

Considering the potentially negative effect of bacterial cell mass at the high inclusion level in the nursery diet, we conducted the experiment first at the low inclusion level of LCM from 0 to 1%, and then conducted the experiment at the high inclusion level of LCM from 0 to 3.5 or 2.2% for complete replacement of synthetic lysine. In the current study, an increase in the dietary level of LCM from 0 to 1% substituted with L-lysine·HCl did not affect the growth performance of weaning pigs, whereas 100% replacement of L-lysine·HCl with LCM at 3.5% and 2.2% for phase 1 and phase 2, respectively, showed negative impacts on the ADG and G–F ratio of weaning pigs. The results of growth performance in the current study were in agreement with the studies of Øverland et al. [21] and Zhang et al. [10], who observed that a higher level of SCP supplementation led to worse growth performance in weaning pigs. Zhang et al. [10] reported that there was no difference in the ADG and feed efficiency among weaning pigs fed the diet with 5% fish meal and 2.5% LCM produced by *Corynebacterium glutamicum*, whereas the feed efficiency and ADG were reduced in pigs fed the diet with 5% of LCM compared to those of weaning pigs fed the diet with 5% fish meal. Moreover, Øverland et al. [21] reported that replacing SBM with 5 to 15% bacterial protein meal produced mainly by *Methylococcus capsulatus* linearly decreased the growth performance of weaning pigs. The reduced growth performance of weaning pigs due to an increase in the supplementation of cell mass coproducts may be explained by the decreased digestibility of CP due to the increased nondigestible cell walls of the microorganisms. Piao et al. [12] reported that an increased inclusion level of LCM from 1 to 5% decreased CP digestibility in broiler chickens for the starter period (0 to 3 weeks). Zhang et al. [10] reported that CP digestibility for weaning pigs fed a diet with 5% SCP (prosine: 81.28%; protine: 81.17%) was less than that for weaning pigs fed a diet with 2.5% SCP (prosine: 83.74%; protine: 84.17%), which was because the higher level of SCP reduced the villus height to crypt depth ratio for the jejunum (prosine: 1.95 to 1.77; protine: 1.91 to 1.72) and ileum (prosine: 2.34 to 2.09; protine: 2.39 to 2.18) in weaning pigs. Thus, it appears that LCM could be included in weaning pigs’ diets up to 2.8% for phase 1 and 1.76% for phase 2 without a detrimental effect on the growth performance of weaning pigs.

In the current study, an increasing dietary level of LCM up to 1% did not affect the fecal score of weaning pigs, and an increasing dietary level of LCM up to 3.5% and 2.2% for phase 1 and phase 2 did not affect the frequency of diarrhea in pigs for the overall period. Diarrhea in weaning pigs is caused by many stress factors associated with weaning and the proliferation of enterotoxigenic *E. coli*, which may negatively influence the response of the immune system and intestinal gut dysfunction in weaning pigs [22,23]. The LCM was produced through the procedure of drying and screening to prevent bacterial contamination and mycotoxin problems. Thus, partial or complete replacement of L-lysine·HCl with LCM in weaning pigs’ diets did not cause diarrhea in the weaning pigs.

In general, BUN is the indicator for the determination of nitrogen utilization by pigs, and it is related to the intake of protein, protein quality, and amino acid balance [24,25]. The BUN concentration in pigs increased due to increases in dietary protein or AA levels or less utilization for protein or AAs [26,27]. Moreover, the BUN concentration can be used to estimate the dietary lysine content required to maximize the utilization of total nitrogen in pigs [24]. It should be noted that LCM has greater AA content, except for lysine, than L-lysine·HCl. In the current study, we increased the inclusion level of LCM in the diets while replacing L-lysine·HCl, resulting in an increase of dietary AAs except for Lys, Met, and Thr. Thus, the linearly increased BUN concentrations in experiment 2 resulting from the increase of dietary LCM with replacement of L-lysine·HCl could be attributed to the fact that the LCM contained lysine with other AAs. IGF-1, as a growth hormone, plays an important role in growth and differentiation for body tissue, controlling the development of the cardiovascular system and skeletal maturation in animals [28]. Nutritional factors are the major determinants of animal growth and are associated with the expression of growth-regulatory genes [29]. In addition, Liao et al. [30] reported that the blood IGF-1 concentration in pigs decreased as the dietary lysine level decreased, implying that the blood IGF-1 concentration could be affected by the lysine content in the swine diet. In the current study, partial replacement of L-lysine·HCl with LCM from 0 to 1% did not affect the blood IGF-1 concentration and ADG in pigs, whereas 100% replacement of L-lysine·HCl with LCM showed no difference in the blood IGF-1 concentration but a decrease in the ADG. Thus, it appears that increasing dietary LCM with replacement of L-lysine·HCl had no influence on the dietary lysine intake of pigs, and there are factors other than dietary lysine content that might influence the effect of dietary LCM on the growth performance of weaning pigs.

The increased serum concentrations of cortisol and insulin for 5 weeks in weaning pigs fed the diets with 0 to 1% LCM observed in the current study (experiment 1) may have partly resulted from the intake of unidentified stressors from the LCM. Because the LCM was produced from cell sludge, which is a byproduct from cell separation after microorganism fermentation during lysine production, it may contain fermentation products, substrate residue, and microbial carcass. The secretion of cortisol was increased in the bodies of pigs when they became stressed from a change in diet or environment, causing an immune response related to humoral and cellular immunity [31,32]. To alleviate the stress response in the body, secreted cortisol promotes glucose metabolism and increases insulin secretion in the body. Cell mass made from bacteria has been found to be associated with some problems including a high concentration of ribonucleic acids, which elevates the uric acid concentration in the blood, causing kidney stones [19]. In addition, it has been noted that the consumption of foreign proteins can cause skin reactions and gastrointestinal reactions such as nausea and vomiting [33]. However, we observed a tendency of linearly decreased serum cortisol concentration in weaning pigs fed the diet with LCM from 0 to 3.5% (experiment 2). Since the mechanisms by which dietary LCM could affect the serum cortisol concentration have not been well established, there is a need to establish the mechanisms by which dietary LCM affects the serum cortisol concentration in weaning pigs. Serum IgG and IgA are the major components of the humoral immunity of pigs. IgG plays an essential role in the systemic immune response, and IgA plays an important role in the immune response of mucous membranes [34,35]. The production of immunoglobulins is induced by the presence of feed or microbial antigens [36]. Thus, the linear decrease in serum IgG concentration due to an increase in the dietary level of LCM implies that increasing the level of dietary LCM reduced the immune response in weaning pigs.

## 5. Conclusions

In conclusion, partial replacement of L-lysine·HCl with 0 to 1% LCM had no adverse effects on the growth performance or diarrhea incidence in weaning pigs. However, the growth performance of weaning pigs was decreased by an increase in the dietary level of LCM at 3.5% for phase 1 and 2.2% for phase 2 with 100% replacement of L-lysine·HCl in the weaning pigs’ diet. Nevertheless, a higher inclusion level of LCM with replacement of L-lysine·HCl linearly decreased the serum IgG concentration in weaning pigs. Therefore, 80% replacement of L-lysine·HCl with LCM 2.8% and 1.76% for phases 1 and 2, respectively, could be applied in weaning pigs’ diets without detrimental effects on the growth performance, diarrhea incidence, or immune response of weaning pigs.

## Figures and Tables

**Table 1:** Analyzed nutrient contents of lysine cell mass (as-is basis).

Item	Lysine Cell Mass
Moisture, %	8.12
Crude ash, %	5.63
Crude protein, %	67.97
Ether extract, %	2.16
Ca, %	0.08
*p*, %	0.40
Non-protein nitrogen, %	ND ^(1)^
Total amino acids, %	44.49
Indispensable amino acids, %	
Arg	2.38
His	0.62
Ile	1.40
Leu	3.43
Lys	9.46
Met	1.06
Phe	1.73
Thr	2.31
Val	1.54
Dispensable amino acids, %	
Ala	4.17
Asp	4.50
Cys	0.19
Glu	5.72
Gly	2.00
Pro	0.99
Ser	1.78
Tyr	1.22

^(1)^ Not detected.

**Table 2:** The diet formulation and chemical composition of experimental diets for experiment 1 (phase 1).

Item	LCM, % ^(1)^				
	0	0.25	0.50	0.75	1.00
Ingredient, % as fed					
Ground corn	33.28	33.39	33.50	33.60	33.71
Soybean meal, 45%	34.97	34.61	34.25	33.90	33.54
Barley	15.00	15.00	15.00	15.00	15.00
Whey powder	1.00	1.00	1.00	1.00	1.00
Lactose	11.00	11.00	11.00	11.00	11.00
Soybean oil	1.25	1.27	1.29	1.31	1.32
L-Lysine·HCl, 78% ^(2)^	0.27	0.25	0.23	0.21	0.19
DL-Methionine, 80%	0.08	0.08	0.08	0.08	0.08
L-Threonine, 99%	0.07	0.07	0.07	0.07	0.07
Lysine cell mass ^(3)^	0.00	0.25	0.50	0.75	1.00
MDCP ^(4)^	1.45	1.45	1.45	1.45	1.45
Limestone	1.03	1.03	1.03	1.03	1.03
Vit. premix ^(5)^	0.10	0.10	0.10	0.10	0.10
Min. premix ^(6)^	0.10	0.10	0.10	0.10	0.10
Salt	0.30	0.30	0.30	0.30	0.30
Zinc oxide, 77.3%	0.10	0.10	0.10	0.10	0.10
Total	100.00	100.00	100.00	100.00	100.00
Calculated chemical composition					
ME, kcal/kg	3265	3265	3265	3265	3265
Crude protein, %	20.56	20.56	20.56	20.56	20.56
Lysine, %	1.35	1.35	1.35	1.35	1.35
Methionine, %	0.35	0.35	0.35	0.35	0.35
Threonine, %	0.86	0.86	0.86	0.86	0.86
Ca, %	0.80	0.80	0.80	0.80	0.80
Total P, %	0.65	0.65	0.65	0.65	0.65

^(1)^ Corn–soybean meal-based diets containing 0, 0.25, 0.50, 0.75, or 1.00% lysine cell mass (LCM) with a substitution of 0 to 0.8% L-lysine·HCl for phase 1 diets; ^(2)^ L-lysine·HCl was provided by Daesang Inc. (Seoul, Korea); ^(3)^ lysine cell mass was provided by Daesang Inc. (Seoul, Korea); ^(4)^ mono-dicalcium phosphate; ^(5)^ provided the following per kilogram of diet: vitamin A, 8000 IU; vitamin D_3_, 1800 IU; vitamin E, 60 IU; thiamine, 2 mg; riboflavin, 7 mg; calcium pantothenic acid, 25 mg; niacin, 27 mg; pyridoxine, 3 mg; biotin, 0.2 mg; folic acid, 1 mg; vitamin B_12_, 0.03 mg; ^(6)^ provided the following per kilogram of diet: Se, 0.3 mg; I, 1 mg; Mn, 51.6 mg; CuSO_4_, 105 mg; Fe, 150 mg; Zn, 72 mg; Co, 0.5 mg.

**Table 3:** The diet formulation and chemical composition of experimental diets for experiment 1 (phase 2).

Item	LCM, % ^(1)^				
	0	0.25	0.50	0.75	1.00
Ingredient, % as fed					
Ground corn	44.89	45.00	45.12	45.22	45.32
Soybean meal, 45%	29.99	29.63	29.26	28.91	28.56
Barley	15.00	15.00	15.00	15.00	15.00
Whey powder	1.00	1.00	1.00	1.00	1.00
Lactose	5.00	5.00	5.00	5.00	5.00
Soybean oil	1.25	1.27	1.29	1.31	1.33
L-Lysine·HCl, 78% ^(2)^	0.16	0.15	0.13	0.11	0.09
DL-Methionine, 80%	0.03	0.03	0.03	0.03	0.03
L-Threonine, 99%	0.01	0.01	0.01	0.01	0.01
Lysine cell mass ^(3)^	0.00	0.25	0.50	0.75	1.00
MDCP ^(4)^	1.22	1.22	1.22	1.22	1.22
Limestone	0.89	0.89	0.89	0.89	0.89
Vit. premix ^(5)^	0.10	0.10	0.10	0.10	0.10
Min. premix ^(6)^	0.10	0.10	0.10	0.10	0.10
Salt	0.30	0.30	0.30	0.30	0.30
Zinc oxide, 77.3%	0.05	0.05	0.05	0.05	0.05
Total	100.00	100.00	100.00	100.00	100.00
**Calculated chemical composition**					
ME, kcal/kg	3265	3265	3265	3265	3265
Crude protein, %	18.88	18.88	18.88	18.88	18.88
Lysine, %	1.15	1.15	1.15	1.15	1.15
Methionine, %	0.30	0.30	0.30	0.30	0.30
Threonine, %	0.75	0.75	0.75	0.75	0.75
Ca, %	0.70	0.70	0.70	0.70	0.70
Total P, %	0.60	0.60	0.60	0.60	0.60

^(1)^ Corn–soybean meal-based diets containing 0, 0.25, 0.50, 0.75, or 1.00% LCM with a substitution of 0 to 0.07% L-lysine·HCl for phase 2 diets; ^(2)^ L-Lysine·HCl was provided by Daesang Inc. (Seoul, Korea); ^(3)^ lysine cell mass was provided by Daesang Inc. (Seoul, Korea); ^(4)^ mono-dicalcium phosphate; ^(5)^ provided the following per kilogram of diet: vitamin A, 8000 IU; vitamin D_3_, 1800 IU; vitamin E, 60 IU; thiamine, 2 mg; riboflavin, 7 mg; calcium pantothenic acid, 25 mg; niacin, 27 mg; pyridoxine, 3 mg; biotin, 0.2 mg; folic acid, 1 mg; vitamin B_12_, 0.03 mg; ^(6)^ provided the following per kilogram of diet: Se, 0.3 mg; I, 1 mg; Mn, 51.6 mg; CuSO_4_, 105 mg; Fe, 150 mg; Zn, 72 mg; Co, 0.5 mg.

**Table 4:** The diet formulation and chemical composition of experimental diets for experiment 2 (phase 1).

Item	Replacement of L-Lysine·HCl with LCM, % ^(1)^					
	0	20	40	60	80	100
Ingredient, % as fed						
Ground corn	33.28	33.59	33.86	34.15	34.40	34.70
Soybean meal, 45%	34.97	33.96	32.98	31.98	31.00	30.01
Barley	15.00	15.00	15.00	15.00	15.00	15.00
Whey powder	1.00	1.00	1.00	1.00	1.00	1.00
Lactose	11.00	11.00	11.00	11.00	11.00	11.00
Soybean oil	1.25	1.30	1.36	1.42	1.49	1.54
L-Lysine·HCl, 78% ^(2)^	0.27	0.22	0.16	0.11	0.05	0.00
DL-Methionine, 80%	0.08	0.08	0.08	0.08	0.07	0.07
L-Threonine, 99%	0.07	0.07	0.07	0.07	0.07	0.07
Lysine cell mass ^(3)^	0.00	0.70	1.40	2.10	2.80	3.50
MDCP ^(4)^	1.45	1.45	1.46	1.47	1.49	1.49
Limestone	1.03	1.03	1.03	1.03	1.03	1.03
Vit. premix ^(5)^	0.10	0.10	0.10	0.10	0.10	0.10
Min. premix ^(6)^	0.10	0.10	0.10	0.10	0.10	0.10
Salt	0.30	0.30	0.30	0.30	0.30	0.30
Zinc oxide, 77.3%	0.10	0.10	0.10	0.10	0.10	0.10
Total	100.00	100.00	100.00	100.00	100.00	100.00
Calculated chemical composition						
ME, kcal/kg	3265	3265	3265	3265	3265	3265
Crude protein, %	20.56	20.56	20.56	20.56	20.56	20.56
Lysine, %	1.35	1.35	1.35	1.35	1.35	1.35
Methionine, %	0.35	0.35	0.35	0.35	0.35	0.35
Threonine, %	0.86	0.86	0.86	0.86	0.86	0.86
Ca, %	0.80	0.80	0.80	0.80	0.80	0.80
Total P, %	0.65	0.65	0.65	0.65	0.65	0.65

^(1)^ Corn–soybean meal-based diets containing 0, 0.7, 1.4, 2.1, 2.8, or 3.5% lysine cell mass (LCM) with a substitution of 0 to 0.27% L-lysine·HCl for phase 1 diets; ^(2)^ L-lysine·HCl was provided by Daesang Inc. (Seoul, Korea); ^(3)^ lysine cell mass was provided by Daesang Inc. (Seoul, Korea); ^(4)^ mono-dicalcium phosphate; ^(5)^ provided the following per kilogram of diet: vitamin A, 8000 IU; vitamin D_3_, 1800 IU; vitamin E, 60 IU; thiamine, 2 mg; riboflavin, 7 mg; calcium pantothenic acid, 25 mg; niacin, 27 mg; pyridoxine, 3 mg; biotin, 0.2 mg; folic acid, 1 mg; vitamin B_12_, 0.03 mg; ^(6)^ provided the following per kilogram of diet: Se, 0.3 mg; I, 1 mg; Mn, 51.6 mg; CuSO_4_, 105 mg; Fe, 150 mg; Zn, 72 mg; Co, 0.5 mg.

**Table 5:** The diet formulation and chemical composition of experimental diets for experiment 2 (phase 2).

Item	Replacement of L-Lysine·HCl with LCM, % ^(1)^					
	0	20	40	60	80	100
Ingredient, % as fed						
Ground corn	44.93	45.10	45.24	45.44	45.62	48.80
Soybean meal, 45%	29.95	29.33	28.85	28.11	27.48	26.87
Barley	15.00	15.00	15.00	15.00	15.00	15.00
Whey powder	1.00	1.00	1.00	1.00	1.00	1.00
Lactose	5.00	5.00	5.00	5.00	5.00	5.00
Soybean oil	1.24	1.28	1.31	1.36	1.40	1.43
L-Lysine·HCl, 78% ^(2)^	0.18	0.15	0.11	0.07	0.04	0.00
DL-Methionine, 80%	0.04	0.03	0.03	0.03	0.03	0.02
L-Threonine, 99%	0.01	0.01	0.00	0.00	0.00	0.00
Lysine cell mass ^(3)^	0.00	0.44	0.88	1.32	1.76	2.20
MDCP ^(4)^	1.20	1.22	1.22	1.23	1.24	1.24
Limestone	0.90	0.89	0.89	0.89	0.89	0.89
Vit. premix ^(5)^	0.10	0.10	0.10	0.10	0.10	0.10
Min. premix ^(6)^	0.10	0.10	0.10	0.10	0.10	0.10
Salt	0.30	0.30	0.30	0.30	0.30	0.30
Zinc oxide, 77.3%	0.05	0.05	0.05	0.05	0.05	0.05
Total	100.00	100.00	100.00	100.00	100.00	100.00
Calculated chemical composition						
ME, kcal/kg	3265	3265	3265	3265	3265	3265
Crude protein, %	18.88	18.88	18.88	18.88	18.88	18.88
Lysine, %	1.16	1.16	1.16	1.16	1.16	1.16
Methionine, %	0.30	0.30	0.30	0.30	0.30	0.30
Threonine, %	0.74	0.74	0.74	0.74	0.74	0.74
Ca, %	0.70	0.70	0.70	0.70	0.70	0.70
Total P, %	0.60	0.60	0.60	0.60	0.60	0.60

^(1)^ Corn–soybean meal-based diets containing 0, 0.25, 0.50, 0.75, or 1.00% LCM with a substitution of 0 to 0.07% L-lysine·HCl for phase 2 diets; ^(2)^ L-Lysine·HCl was provided by Daesang Inc. (Seoul, Korea); ^(3)^ lysine cell mass was provided by Daesang Inc. (Seoul, Korea); ^(4)^ mono-dicalcium phosphate; ^(5)^ provided the following per kilogram of diet: vitamin A, 8000 IU; vitamin D_3_, 1800 IU; vitamin E, 60 IU; thiamine, 2 mg; riboflavin, 7 mg; calcium pantothenic acid, 25 mg; niacin, 27 mg; pyridoxine, 3 mg; biotin, 0.2 mg; folic acid, 1 mg; vitamin B_12_, 0.03 mg; ^(6)^ provided the following per kilogram of diet: Se, 0.3 mg; I, 1 mg; Mn, 51.6 mg; CuSO_4_, 105 mg; Fe, 150 mg; Zn, 72 mg; Co, 0.5 mg.

**Table 6:** Effects of LCM supplementation levels on growth performance in weaning pigs (exp. 1).

Item ^(1)^	LCM, % ^(2)^					SEM ^(3)^	*p*-Value ^(4)^		
	0	0.25	0.50	0.75	1.00		Diet	Lin.	Quad.
Body weight, kg									
Initial	6.89	6.89	6.89	6.89	6.89	0.229	-	-	-
2 week	9.52	9.21	9.75	9.44	9.55	0.284	0.62	0.71	0.99
5 week	18.01	18.11	18.34	17.85	18.76	0.467	0.87	0.56	0.72
Average daily gain, g/d									
0–2 weeks	203	179	220	195	205	7.9	0.62	0.72	0.98
2–5 weeks	404	431	404	396	438	11.0	0.85	0.64	0.56
0–5 weeks	327	331	337	321	349	8.6	0.84	0.56	0.71
Average daily feed intake, g/d									
0–2 weeks	333	342	355	342	335	11.8	0.95	0.93	0.46
2–5 weeks	791	798	772	749	826	19.8	0.86	0.88	0.38
0–5 weeks	616	623	613	593	638	15.5	0.39	0.88	0.57
Gain:feed ratio									
0–2 weeks	0.608	0.518	0.625	0.575	0.611	0.015	0.07	0.45	0.33
2–5 weeks	0.511	0.542	0.524	0.532	0.530	0.008	0.87	0.72	0.63
0–5 weeks	0.531	0.531	0.552	0.544	0.546	0.007	0.43	0.44	0.61

^(1)^ Least squares means of 4 replications per treatment; ^(2)^ corn–soybean meal-based diets containing 0, 0.25, 0.50, 0.75, or 1.00% LCM with a substitution of 0 to 0.8% L-lysine·HCl for phase 1 diets and 0 to 0.07% L-lysine·HCl for phase 2 diets; ^(3)^ standard error of the mean; ^(4)^ Lin.: linear effect, Quad.: quadratic effect.

**Table 7:** Effects of LCM supplementation levels on fecal score in weaning pigs (exp. 1).

Item ^(1)^	LCM, % ^(2)^					SEM ^(3)^	*p*-Value ^(4)^		
	0	0.25	0.50	0.75	1.00		Diet	Lin.	Quad.
Fecal score ^(5)^									
0–2 weeks	2.15	2.46	1.77	1.92	2.38	0.124	0.08	0.91	0.16
2–5 weeks	1.91	2.37	2.27	1.91	2.05	0.098	0.11	0.67	0.08
0–5 weeks	2.00 ^b^	2.40 ^a^	2.09 ^ab^	1.91 ^b^	2.17 ^ab^	0.063	0.03	0.70	0.75

^ab^ Within a row, means without a common superscript differ (*p* < 0.05); ^(1)^ least squares means of 4 observations per treatment; ^(2)^ corn–soybean meal-based diets containing 0, 0.25, 0.50, 0.75, or 1.00% LCM with a substitution of 0 to 0.8% L-lysine·HCl for phase 1 diets and 0 to 0.07% L-lysine·HCl for phase 2 diets; ^(3)^ standard error of the mean; ^(4)^ Lin.: linear effect, Quad.: quadratic effect; ^(5)^ fecal score: 1 = firm and shaped feces, 2 = normal and formed feces, 3 = soft and wet feces, 4 = mild diarrhea, and 5 = watery diarrhea.

**Table 8:** Effects of LCM supplementation levels on blood profiles in weaning pigs (exp. 1).

Item ^(1)^	LCM, % ^(2)^					SEM ^(3)^	*p*-Value ^(4)^		
	0	0.25	0.50	0.75	1.00		Diet	Lin.	Quad.
Blood urea nitrogen, mg/dL									
2 weeks	14.00	14.33	14.75	13.27	16.82	0.567	0.37	0.23	0.31
5 weeks	17.65	13.05	16.72	13.92	13.63	0.799	0.27	0.21	0.74
Insulin-like growth factor-1, ng/mL									
2 weeks	99.63	94.73	90.98	72.77	101.53	5.707	0.55	0.67	0.30
5 weeks	108.70	96.33	98.65	137.47	123.18	7.024	0.31	0.17	0.58
Cortisol, μg/dL									
2 weeks	4.05	3.68	3.90	5.30	3.98	0.387	0.68	0.62	0.84
5 weeks	3.32 ^c^	8.90 ^a^	5.73 ^bc^	7.08 ^ab^	7.62 ^ab^	0.485	0.02	0.01	0.07
Insulin, μU/mL									
2 weeks	0.65	0.53	0.71	0.98	0.82	0.076	0.45	0.14	0.98
5 weeks	0.48	0.56	0.65	0.73	1.02	0.082	0.17	0.05	0.57
Immunoglobulin A, mg/mL									
2 weeks	0.33	0.37	0.34	0.33	0.30	0.019	0.90	0.51	0.56
5 weeks	0.47	0.44	0.38	0.38	0.38	0.027	0.80	0.25	0.65
Immunoglobulin G, mg/mL									
2 weeks	2.72	3.40	2.87	2.87	2.72	0.150	0.63	0.63	0.39
5 weeks	4.45 ^ab^	3.83 ^ab^	4.91 ^a^	3.02 ^c^	3.34 ^bc^	0.200	0.01	0.02	0.43

^abc^ Within a row, means without a common superscript differ (*p* < 0.05); ^(1)^ least squares means of 6 observations per treatment; ^(2)^ corn–soybean meal-based diets containing 0, 0.25, 0.50, 0.75, or 1.00% LCM with a substitution of 0 to 0.8% L-lysine·HCl for phase 1 diets and 0 to 0.07% L-lysine·HCl for phase 2 diets; ^(3)^ standard error of the mean; ^(4)^ Lin.: linear effect, Quad.: quadratic effect.

**Table 9:** Effects of LCM supplementation levels on growth performance in weaning pigs (exp. 2).

Item ^(1)^	Replacement of L-Lysine·HCl with LCM, % ^(2)^						SEM ^(3)^	*p*-Value ^(4)^		
	0	20	40	60	80	100		Diet	Lin.	Quad.
Body weight, kg										
Initial	6.44	6.44	6.44	6.44	6.44	6.43	0.280	-	-	-
2 week	8.81	8.26	8.49	8.20	9.43	8.28	0.436	0.65	0.87	0.92
5 week	17.30	16.64	16.99	16.97	17.92	13.36	0.779	0.12	0.11	0.09
Average daily gain, g/d										
0–2 weeks	170	130	146	126	214	132	16.5	0.65	0.86	0.91
2–5 weeks	404 ^a^	399 ^a^	405 ^a^	418 ^a^	404 ^a^	242 ^b^	20.5	0.01	<0.01	<0.01
0–5 weeks	310	291	302	301	328	198	16.8	0.12	0.11	0.09
Average daily feed intake, g/d										
0–2 weeks	306	272	297	240	305	303	16.9	0.51	0.96	0.12
2–5 weeks	861	798	847	843	782	769	30.7	0.91	0.39	0.90
0–5 weeks	639	588	627	602	591	583	23.8	0.96	0.46	0.80
Gain:feed ratio										
0–2 weeks	0.546	0.476	0.461	0.516	0.704	0.399	0.0424	0.55	0.85	0.44
2–5 weeks	0.467 ^a^	0.504 ^a^	0.484 ^a^	0.503 ^a^	0.519 ^a^	0.313 ^b^	0.0212	0.03	0.06	<0.01
0–5 weeks	0.483 ^bc^	0.496 ^b^	0.481 ^b^	0.505 ^ab^	0.557 ^a^	0.335 ^c^	0.0209	0.04	0.16	0.01

^abc^ Within a row, means without a common superscript differ (*p* < 0.05); ^(1)^ least squares means of 4 replications per treatment; ^(2)^ corn–soybean meal-based diets containing 0 to 3.5% of LCM for phase 1 and 0 to 2.2% of LCM for phase 2 with a substitution of L-lysine·HCl from 0 to 100%; ^(3)^ standard error of the mean; ^(4)^ Lin.: linear effect, Quad.: quadratic effect.

**Table 10:** Effects of LCM supplementation levels on blood profiles in weaning pigs (exp. 2).

Item ^(1)^	Replacement of L-Lysine·HCl with LCM, % ^(2)^						SEM ^(3)^	*p*-Value ^(4)^		
	0	20	40	60	80	100		Diet	Lin.	Quad.
Blood urea nitrogen, mg/dL										
2 weeks	15.83	16.75	14.35	19.03	16.37	13.88	0.632	0.28	0.99	0.02
5 weeks	9.77 ^b^	12.53 ^a^	11.58 ^a^	9.72 ^b^	13.25 ^a^	14.90 ^a^	0.443	<0.01	<0.01	0.04
Insulin-like growth factor-1, ng/mL										
2 weeks	39.07	65.05	64.18	54.08	60.12	63.77	5.464	0.72	0.54	0.84
5 weeks	100.27	110.08	80.72	88.47	129.33	86.58	7.044	0.46	0.72	0.48
Cortisol, μg/dL										
2 weeks	6.17	3.62	5.25	4.73	4.03	3.72	0.493	0.26	0.09	0.60
5 weeks	1.92	5.00	6.03	4.80	3.10	5.60	0.289	0.14	0.61	0.95
Insulin, μU/mL										
2 weeks	0.98 ^a^	0.85 ^ab^	0.60 ^b^	0.90 ^ab^	0.40 ^c^	0.73 ^ab^	0.064	0.06	0.18	0.95
5 weeks	0.80	0.60	0.50	0.52	0.72	0.87	0.071	0.47	0.38	0.15
Immunoglobulin A, mg/mL										
2 weeks	0.35	0.22	0.34	0.33	0.28	0.27	0.025	0.65	0.48	0.74
5 weeks	0.58	0.50	0.55	0.58	0.50	0.52	0.038	0.98	0.70	0.84
Immunoglobulin G, mg/mL										
2 weeks	2.54 ^a^	2.28 ^ab^	2.02 ^b^	1.93 ^b^	2.03 ^b^	2.08 ^b^	0.057	0.01	0.02	0.12
5 weeks	4.96	3.84	4.92	4.19	3.57	4.33	0.182	0.20	0.11	0.11

^abc^ Means in a same row with different superscript letters significantly differ (*p* < 0.05); ^(1)^ least squares means of 6 observations per treatment; ^(2)^ corn–soybean meal-based diets containing 0 to 3.5% of LCM for phase 1 and 0 to 2.2% of LCM for phase 2 with a substitution of L-lysine·HCl from 0 to 100%; ^(3)^ standard error of the mean; ^(4)^ Lin.: linear effect, Quad.: quadratic effect.

## Data Availability

The data presented in this study are available on request from the corresponding author.
